# Transdermal Drug Delivery: Innovative Pharmaceutical Developments Based on Disruption of the Barrier Properties of the stratum corneum

**DOI:** 10.3390/pharmaceutics7040438

**Published:** 2015-10-22

**Authors:** Ahlam Zaid Alkilani, Maelíosa T.C. McCrudden, Ryan F. Donnelly

**Affiliations:** 1 School of Pharmacy, 97 Lisburn Road, Queens University Belfast, Belfast BT9 7BL, Northern Ireland, UK; E-Mails: m.mccrudden@qub.ac.uk (M.T.C.M.); R.Donnelly@qub.ac.uk (R.F.D.); 2 Faculty of Pharmacy, Zarqa University, Zarqa 132222, Jordan

**Keywords:** transdermal, drug delivery, velocity based device, ultrasound, thermal ablation, mechanical and electrical approaches, microneedle

## Abstract

The skin offers an accessible and convenient site for the administration of medications. To this end, the field of transdermal drug delivery, aimed at developing safe and efficacious means of delivering medications across the skin, has in the past and continues to garner much time and investment with the continuous advancement of new and innovative approaches. This review details the progress and current status of the transdermal drug delivery field and describes numerous pharmaceutical developments which have been employed to overcome limitations associated with skin delivery systems. Advantages and disadvantages of the various approaches are detailed, commercially marketed products are highlighted and particular attention is paid to the emerging field of microneedle technologies.

## 1. Introduction

The most common routes of drug delivery are the oral and parenteral routes with the majority of small molecule drugs conventionally delivered orally [1,2]. The oral route has the advantage of pre-determined doses, portability and patient self-administration. For these reasons, the oral route remains the most convenient means of delivering medications [3,4]. However, most therapeutic peptides or proteins are not delivered by the oral route, due to rapid degradation in the stomach and size-limited transport across the epithelium [5]. The primary mode of administering macromolecules is therefore via injection [1,5,6] which is not without limitations, such as the invasive nature of injections eliciting pain and lower acceptance/compliance by patients, in addition to the requirement for administration by a trained administrator [5,6,7]. Rationally, the conventional routes of medication delivery have many inherent limitations which could potentially be overcome by advanced drug delivery methodologies such as transdermal drug delivery (TDD).

## 2. Transdermal Drug Delivery (TDD)

TDD is a painless method of delivering drugs systemically by applying a drug formulation onto intact and healthy skin [2,5]. The drug initially penetrates through the stratum corneum and then passes through the deeper epidermis and dermis without drug accumulation in the dermal layer. When drug reaches the dermal layer, it becomes available for systemic absorption via the dermal microcirculation [8,9]. TDD has many advantages over other conventional routes of drug delivery [10,11,12]. It can provide a non-invasive alternative to parenteral routes, thus circumventing issues such as needle phobia [2]. A large surface area of skin and ease of access allows many placement options on the skin for transdermal absorption [5]. Furthermore, the pharmacokinetic profiles of drugs are more uniform with fewer peaks, thus minimizing the risk of toxic side effects [2]. It can improve patient compliance due to the reduction of dosing frequencies and is also suitable for patients who are unconscious or vomiting, or those who rely on self-administration [13]. TDD avoids pre-systemic metabolism, thus improving bioavailability [2,4]. With reference to the use of the skin as a novel site for vaccination strategies, this organ is known to be replete with dendritic cells in both the epidermal and dermal layers which play a central role in immune responses making TDD an attractive vaccination route for therapeutic proteins and peptides [3,14]. The requirement for an inexpensive and non-invasive means of vaccination, especially in the developing world [3,14,15], has given rise to substantial research focused on the development of simple, needle-free systems such as TDD for vaccination purposes. This theme will be explored further in Section 4.5.2 of this review.

## 3. A Brief Review of Skin Structure

**Figure 1:**
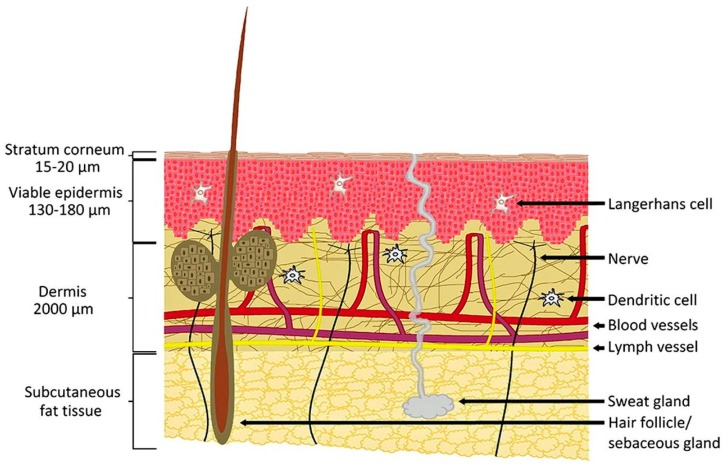
Anatomy of the skin. (Reprinted from [22] with permission. Copyright 2012 Elsevier).

Skin is the most accessible and largest organ of the body with a surface area of 1.7 m^2^, compromising 16% of the total body mass of an average person [16,17,18]. The main function of the skin is to provide a protective barrier between the body and the external environment against microorganisms, the permeation of ultraviolet (UV) radiation, chemicals, allergens and the loss of water [19]. Skin can be divided into three main regions: (1) the outermost layer, the epidermis, which contains the stratum corneum; (2) the middle layer, the dermis and (3) the inner most layer, the hypodermis (Figure 1) [5,20,21].

### 3.1. Epidermis

The epidermis is the outermost layer of the skin and varies in thickness with approximately 0.8 mm on the palms of the hands and soles of the feet [19]. It consists of multi-layered regions of epithelial cells and the viable epidermis is often referred to as the epidermal layers below the stratum corneum [8,19]. The cellular content of the epidermis consists predominantly of keratinocytes (approximately 95% of cells), with other cells of the epidermal layers including melanocytes, Langerhans cells and merkel cells [14].The stratum corneum is the most superficial layer of the epidermis [19,23,24]. It is in direct contact with the external environment and its barrier properties may be partly related to its very high density (1.4 g/cm^3^ in the dry state) and its low hydration of 15%–20% [25]. The cells of the stratum corneum are composed mainly of insoluble keratins (70%) and lipid (20%) [25]. Water in the stratum corneum is associated with keratin in the corneocytes [19,26].

### 3.2. Dermis

The dermis is approximately 2–3 mm thick and consists of collagenous (70%) and elastin fibres which give strength and elasticity to the skin [17]. Blood vessels found in the dermis provide nutrients for both the dermis and epidermis. Nerves, macrophages and lymphatic vessels are also present in the dermis layer, as depicted in Figure 1 [23].

### 3.3. Hypodermis

The hypodermis or subcutaneous layer is the deepest layer of the skin and consists of a network of fat cells [17]. It is the contact layer between the skin and the underlying tissues of the body, such as muscles and bone. Therefore, the major functions of the hypodermis are protection against physical shock, heat insulation and support and conductance of the vascular and neural signals of the skin [27]. Hypodermis-resident fat cells account for approximately 50% of the body’s fat with the other predominant cells of the hypodermis consisting of fibroblasts and macrophages [28].

### 3.4. Drug Penetration Routes

**Figure 2:**
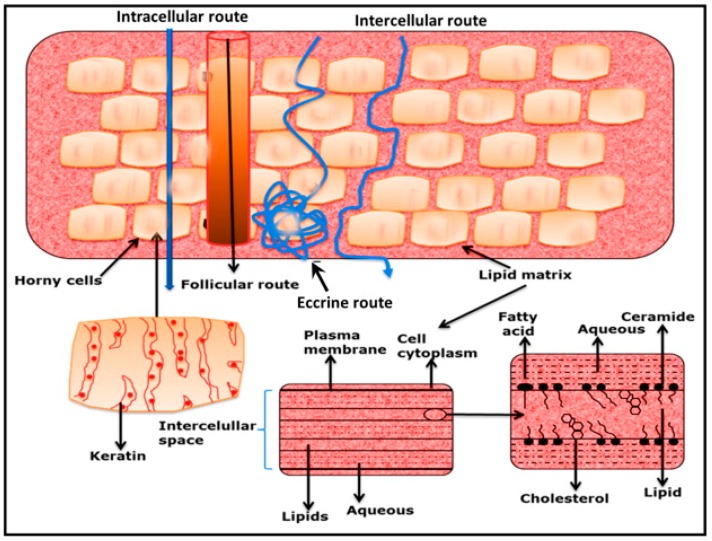
Possible drug penetration routes across human skin. (Reprinted from [30] with permission. Copyright 2012 Elsevier).

There are two possible routes of drug penetration across the intact skin, namely the transepidermal and transappendegeal pathways, which have been diagrammatically presented in Figure 2. The transepidermal pathway involves the passage of molecules through the stratum corneum, an architecturally diverse, multi-layered and multi-cellular barrier. Transepidermal penetration can be termed intra- or inter-cellular [29]. The intra-cellular route through corneocytes, terminally differentiated keratinocytes, allows the transport of hydrophilic or polar solutes. Transport via inter-cellular spaces allows diffusion of lipophilic or non-polar solutes through the continuous lipid matrix. The transappendegeal route involves the passage of molecules through sweat glands and across the hair follicles [5,30].

### 3.5. Kinetics of TDD

An understanding of the kinetics of skin permeation is necessary for development of successful TDD systems. In order to evaluate any TDD, the assessment of percutaneous absorption of molecules is a very important step. Percutaneous absorption is the penetration of substances into various layers of skin and permeation across the skin into the systemic circulation [8,31,32,33]. Percutaneous absorption of molecules is a step wise process involving:

- Penetration: The entry of a substance into a particular layer of the skin;
- Partitioning from the stratum corneum into the aqueous viable epidermis;
- Diffusion through the viable epidermis and into the upper dermis;
- Permeation: The penetration of molecules from one layer into another, which is different both functionally and structurally from the first layer;
- Absorption: The uptake of a substance into the systemic circulation.

**Figure 3:**
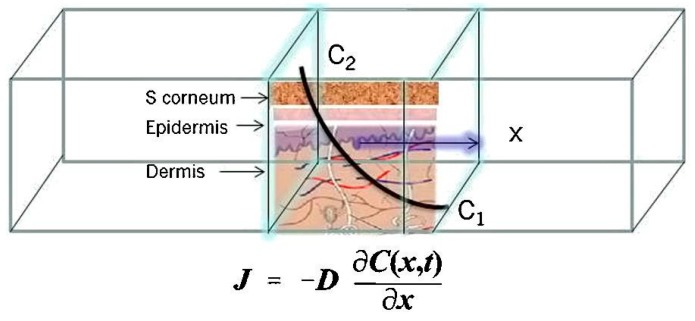
Description of flux across the skin from a transdermal patch where *J* is the molecular flux, *C*_2_ is the concentration of the active molecule in the patch, *C*_1_ is the concentration of the active molecule in the body, *D* is the diffusion coefficient; *L* is the cross sectional thickness of diffusion, and *t* is the diffusion time. The equation indicates Fick’s law of diffusion. (Reprinted from [33] with permission. Copyright 2013 Elsevier).

In delivery systems involving transdermal patches, the drug is stored in a reservoir (reservoir type) or drug dissolved in a liquid or gel-based reservoir (matrix type).The starting point for the evaluation of the kinetics of drug release from a transdermal patch is an estimation of the drug compound’s maximum flux across the skin (flux (*J*)) which is typically expressed in units of μg/cm^2^/h) (Equation 1) (Figure 3). Based on Fick’s law of diffusion, the transport of therapeutic molecules across skin will be maintained until the concentration gradient ceases to exist [33,34,35].

(1) $$J=\;-D\frac{dc}{dl}$$

where *D* is the diffusion coefficient and d*c* / d*t* is the concentration gradient.

Conventional TDD is possible only if the drug possesses certain physiochemical properties. The rate of permeation across the skin (d*Q* / d*t*) is given by [35]:

(2) $$dQ/dt=P\left(C_{d}-C_{r}\right)$$

where *C*_d_ and *C*_r_ are the concentration of the skin penetrant in the donor compartment (*i.e.*, on the surface of stratum corneum*)* and in the receptor compartment (*i.e.*, body), respectively. *P* is the permeability coefficient of the skin tissue to the penetrant.

(3) P=D∗K/L

where *D* is he diffusion coefficient obtained from the permeability coefficient, *P*, the solute partition coefficient, *K*, and *L* is the overall thickness of skin tissues.

From equation (2) it is clear that a constant rate of drug permeation can be obtained only when *C*_d_ >> *C*_r_ *i.e*., the drug concentration at the surface of the stratum corneum. *C_d_* is consistently and substantially greater than the drug concentration in the body *C*_r_. The equation becomes:

(4) $$dQ/dt=P*C_{d}$$

The cumulative amount permeating (*Q*) the barrier with the effective surface area of permeation (*A*) at a given time (*t*) is calculated by using Equation (5) [35]:

(5) $$Q=PAC_{d}t$$

**Figure 4:**
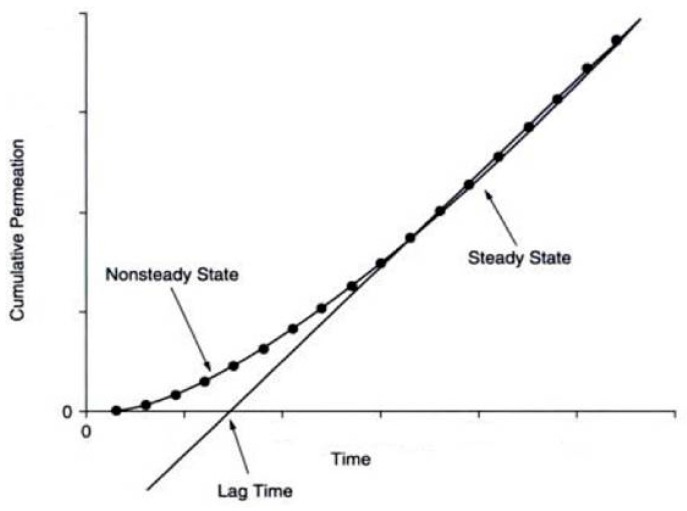
A typical plot of permeation study.

The permeability coefficient (*P*) can be obtained from the slope of a plot of cumulative permeation of diffusant *vs.* time obtained from an experimental permeation study. A typical plot of permeation study is shown in Figure 4.

*L* is the swollen membrane thickness. As shown in Figure 4, the cumulative permeation curve has two portions. The initial portion of the curve represents non-steady state diffusion and the linear portion corresponds to steady state diffusion. The non-steady portion of the curve can be described mathematically by Fick’s second law, while the linear portion can be expressed by Fick’s first law [35]. The time required to reach steady state is called the lag time (*t*Lag). The lag time can be determined by extrapolating the linear portion of permeation *vs.* the time curve to the time axis. With lag time, Equation (6) is rewritten as (7) [35].

(6) $$Q=\left[DKACd\left(t-t_{L}\right)\right]/L$$

(7) $$Q=PACd\left(t-t_{L}\right)$$

The lag time can be calculated by Equation (8) [35]:

(8) $$t_{L}=L^{2}/6D$$

Transdermal systems should be formulated to provide the maximum thermodynamic driving force for passive diffusion across the skin which is saturated with a sufficient payload of the drug to ensure delivery of drugs across the skin. The ability of approved transdermal drugs to penetrate the skin varies widely from the extremely permeable nicotine to compounds, such as buprenorphine and the progestins, which have very low predicted fluxes.

The first transdermal patch approved for systemic delivery in 1979 was a patch for the sustained, three days delivery of scopolamine in the treatment of motion sickness [1,34]. Transdermal delivery is currently restricted to approximately 17 drug molecules that are approved by the US Food and Drug Administration (FDA) (Table 1) [2,4]. The limited number of drug molecules seen in s Table 1 reflects the difficulty of meeting the dual challenge of potent pharmacological activity and the correct physicochemical properties to enable skin penetration [34,36].These approved molecules are all of low molecular weight (MW < 500 Da), a balanced lipophilicity (log *P* = 1–3), and a measurable solubility both in oil and in water because TDD systems require both breaching the lipophilic stratum corneum and resorption into the aqueous central compartment of the systemic circulation [8,34]. Moreover, high pharmacological potency of drug molecules is required to become a feasible candidate for TDD [8,36,37]. The limited permeability of molecules is due to the outermost layer of the skin, the *stratum corneum* [38,39]. This “dead” layer of tissue has the ability to prevent the permeation of foreign compounds including drug molecules and therefore acts as a very effective barrier [39,40]. In order to enhance drug permeation across the skin, a number of chemical and physical methods have been devised [3,5,34].

**Table 1:** Daily dose ranges and selected physicochemical and pharmacokinetic properties of currently approved transdermally delivered drugs. (Reprinted from [34] with permission. Copyright 2014 Elsevier).

Drug (Year of Approval)	Dose/Day (mg)	MW (Da)	Log *P* ^a^	Cl (L/h)	*T*_1/2_ (h)^b^	F (%) ^c^	C_p,eff_ (ng/mL) ^e^
Scopolamine (1979)	0.3	303	0.98	672	2.9	27	0.04
Glyceryl trinitrate (1981)	2.4–15	227	01.62	966	0.04	<1	0.1–5
Clonidine (1984)	0.1–0.3	230	2.42 ± 0.52	13	6–20	95	0.2–2.0
Estradiol (1986)	0.025–0.1	272	4.01	615–790	0.05	3-5	0.04–0.06
Fentanyl (1990)	0.288–2.4	337	4.05	27–75	3–12	32	1.0
Nicotine (1991)	7–21	162	1.17	78	2	30	10–30
Testosterone (1993)	0.3–5	288	3.32		0.17–1.7	<1	10–100
Estradiol & Norethisterone Acetate (1998)	0.025–0.050 0.125–0.250	272 340	4.01 3.99		2–3 6–8 ^d^	3–5 64	0.04–0.07 0.8–1.1
Norelgestromin & EthinylEstradiol (2001)	0.025–0.050 0.125–0.250	327 296	3.90 ± 0.47 3.67		28 17 ^d^	40	0.8 0.05
Estradiol & Levonorgestrel (2003)	0.025–0.050 0.125–0.250	272 312	4.01 3.72 ± 0.49		3 28 ^d^	3-5	0.03–0.05 0.1–0.2
Oxybutynin (2003)	3.9	357	4.02 ± 0.52		2	6	1.0–5.0
Selegeline (2006)	6–12	187	2.90	84	10	10	2.0–3.0
Methylphenidate (2006)	26–80	233	2.15 ± 0.42	20	2–3	5–20	5.0–25
Rotigotine (2007)	1–3	315	4.58 ± 0.72	600	5–7 ^d^	n/a	~1.0
Rivastigmine (2007)	4.6–9.5	250	2.34 ± 0.16	108	1.5	40	~10
Granisetron (2008)	3.1	312	2.55 ± 0.28	33–76 healthy 15–34 patients	4–6 healthy 9–12 patients	60	0.7–9.5
Buprenorphine (2010)	0.12–1.68	468	4.98	55	22–36 ^d^	n/a	0.1–0.4

^a^ Log{octanol-water partition coefficient (*P*)}: either experimental or calculated (mean ± SD) values; ^b^ Terminal half-life post-oral or IV dosing; ^c^ Oral bioavailability; ^d^ Terminal half-life following transdermal delivery; ^e^ Pharmacologically effective plasma concentration.

Due to the aforementioned challenges associated with successful drug permeation across the skin, a number of different, innovative approaches have been explored and developed to overcome these challenges. These will be discussed in the subsequent sections of this review.

## 4. Techniques for Enhancement of Skin Permeabilisation

**Figure 5:**
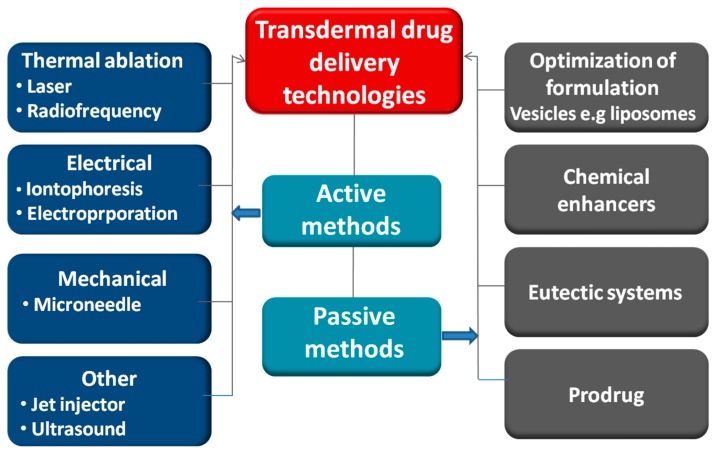
Approaches for enhancing drug transport across the skin.

Technologies used to modify the barrier properties of the stratum corneum can be divided into passive/chemical or active/physical methodologies (Figure 5). Passive methods include the influencing of drug and vehicle interactions and optimization of formulation, in order to modify the stratum corneum structure [29,41,42]. Passive methods are relatively easy to incorporate into transdermal patches such as chemical enhancers and emulsions [43]. However, the main drawback of passive methods may be a lag time in drug release incurred with obvious negative influence on rapid onset drugs, such as insulin.

**Figure 6:**
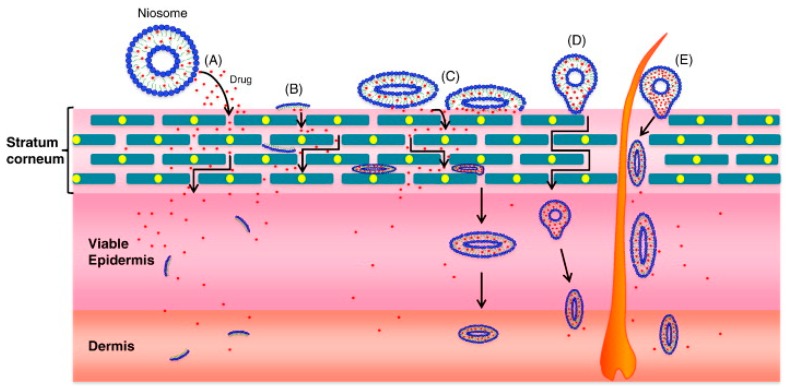
Possible mechanisms of action of surfactant vesicles for dermal and transdermal applications: (**A**) drug molecules are released by niosomes; (**B**) niosome constituents act as penetration enhancer; (**C**) niosome adsorption and/or fusion with stratum corneum; (**D**) intact niosome penetration through the intact skin; (**E**) niosome penetration through hair follicles and/or pilosebaceous units. (Reprinted from [46] with permission. Copyright 2014 Elsevier).

One of the most widely used passive approaches is the use of chemical penetration enhancers which facilitate drug permeation across the skin by increasing drug partitioning into the barrier domain of the stratum corneum, without long-term damage to the skin [11,44]. Penetration enhancers have several mechanisms of action such as: increasing the fluidity of the stratum corneum lipid bilayers, interaction with intercellular proteins, disruption or extraction of intercellular lipids, increase of the drug’s thermodynamic activity and increase in stratum corneum hydration [11,44,45]. Several types of penetration enhancers are known and they can be divided into several groups based on their chemical structure, rather than their mechanism of action [32,44]. Most of these have mixed modes of action so it is difficult to classify them according to this characteristic. Examples of commonly investigated penetration enhancers are alcohols, sulphoxides, azone, pyrrolidones, essential oil, terpenes and terpenoids, fatty acids, water and urea [44,45]. However, the major limitation for penetration enhancers is that their efficacy is often closely correlated with the occurrence of skin irritation [32,45]. Gels have been used in TDD and recent developments in the technology have introduced new variations of semisolid vehicles such as proniosomes and microemulsion gels into the field of penetration enhancers [43]. Proniosomes are non-ionic based surfactant vesicles, they are known as ‘‘dry niosomes’’ because they may require hydration before drug release and permeation through the skin. Proniosomal gels have been used in TDD because they act as penetration enhancers that enhance the drug permeation from the skin barrier [43,46]. Upon hydration proniosomesare converted into niosomes which are capable of diffusing across the stratum corneum and then adhere to the cell surface which causes a high thermodynamic activity gradient of the drug at the vesicle/stratum corneum surface, thus acting as the driving force for the penetration of lipophilic drugs across the skin (Figure 6) [43,46].

Some of the limitations associated with penetration enhancers are poor efficacy and safety. They do not achieve the desired skin disruption and their ability to increase transport across the skin is low and variable [46,47]*.* Regarding safety considerations, penetration enhancers have been shown in a limited number of cases to potentially cause skin irritation including local inflammation, erythema, swelling and dermatitis [47].

The active methods for skin permeabilisation include ultrasound, electrically assisted methods (electroporation and iontophoresis), velocity based devices *(*powder injection, jet injectors), thermal approaches (lasers and radio-frequency heating) and mechanical methodologies such as microneedles (MN) and tape stripping [2,48,49,50,51]. These approaches allow a broader class of drugs to be delivered into the skin. Active methods involve the use of external energy to act as a driving force for drug transport across the skin or by physically disrupting the stratum corneum [48,49]. These techniques greatly expand the range of drugs that can be delivered effectively across the skin. This in turn will significantly enhance the value of the transdermal delivery market and will be increasingly important over the coming years as the number of new drugs of biological origin continues to increase. In addition, active methods also offer more reproducible control over the delivery profiles of the medications, thus overcoming lag times between the application and the drug reaching the systemic circulation when compared to passive methods [11,48]. Some of these active methodologies will be described in detail below.

### 4.1. Ultrasound Devices

Ultrasound is an oscillating sound pressure wave that has long been used for many research areas including physics, chemistry, biology, engineering and others in a wide range of frequencies [2,50]. Ultrasound, sonophoresis, or phonophoresis can be defined as the transport of drugs across the skin by application of ultrasound perturbation at frequencies of 20 kHz–16 MHz which has a sufficient intensity to reduce the resistance of skin [2,5]. The use of ultrasound has resulted in the effective delivery of various different categories and classes of drugs, regardless of their electrical characteristics, by increasing skin permeability. These drugs have included hydrophilic and large molecular weight drugs [39]. However, the mechanism of action is still not clearly understood or characterized [50]. The proposed mechanisms by which ultrasound effects tissues and cells include thermal effects and cavitation effects caused by collapse and acoustic streaming which can be explained as oscillation of cavitation bubbles in the ultrasound field [5]. Ultrasound can increase the temperature of the insonated medium (the skin) by the absorption of the sound waves with a frequency greater than the upper limit of the human hearing range. Obviously, the higher the medium’s absorption coefficient, the higher the increase in temperature and thus the greater the thermal effect [50]. All recent studies point out that cavitation is believed to be the predominant mechanism in the enhancement of TDD via ultrasound treatment [50].

**Figure 7:**
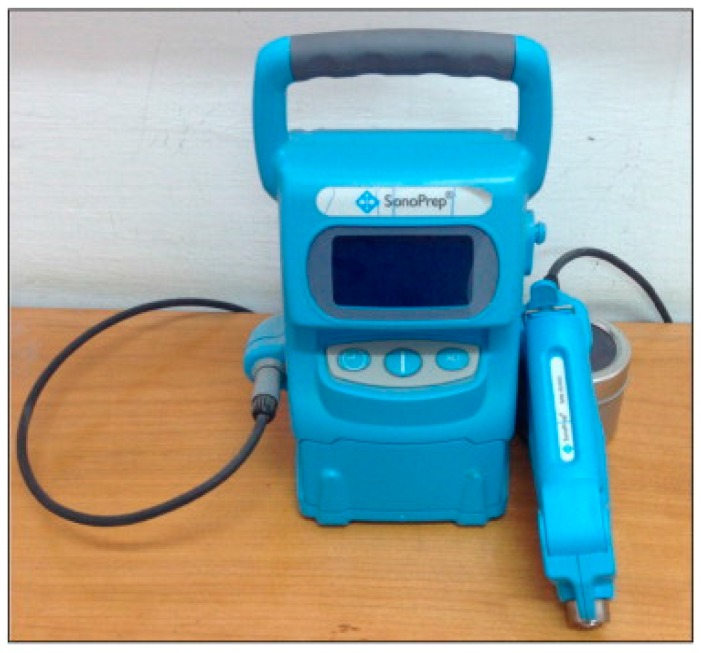
The SonoPrep^®^ ultrasound device (Reprinted from [50] with permission. Copyright 2014 Elsevier).

The concept of ultrasound for use in TDD was initially reported by Fellinger and Schmidt in 1950 for the successful treatment of polyarthritis using hydrocortisone ointment combined with sonophoresis [52,53,54]. However, the first ultrasound device for transdermal application was approved in 2004 by the FDA for the delivery of local dermal anesthesia by the Sontra Medical, SonoPrep^®^ (Figure 7). Since that time, ultrasound has been widely used as a TDD system in the treatment of many other diseases including bone joint diseases and bursitis [2]. Many challenges must be overcome before such devices gain commercial acceptance however. Some of these challenges include: availability of easy-to-use devices; the determination of the duration of treatment required; gaining a full understanding of how the technology functions; broadening of the range of drugs that can be delivered and evaluation of the safety profiles of the devices [5,39,55,56]. Examples of undesirable side effects of ultrasound approaches were observed by Singer *et al.* (1998) when it was shown that low-intensity ultrasound caused minor skin reactions in dogs while high-intensity ultrasound was capable of inducing second-degree burns [56]. Limitations such as this must be overcome before these innovations can garner full acceptance.

### 4.2. Electrical Techniques

#### 4.2.1. Electroporation

The two major means of electrically-facilitated TDD are iontophoresis and electroporation [2,4]. In electroporation, cells are temporarily exposed to high intensities of electric pulses that lead to the formation of aqueous pores in the lipid bilayers of the stratum corneum, thus allowing the diffusion of drugs across skin [5,57,58,59]. The technique was first described by Neumann *et al.* in 1982 [59]. Usage of high voltage pulses (50–500 V) for short times of only one second have been shown to increase transport across the skin for different molecular weight drugs ranging from small e.g., fentanyl, timolol [60,61], orcalcein [62], to high molecular weight drugs such as LHRH, calcitonin, heparin or FITC–dextran with molecular weights up to 40 kDa [58,63,64,65,66]. However, the main drawbacks are the lack of quantitative delivery, cell death with high fields and potential damage to labile drugs, e.g., those of protein origin [57,67].

#### 4.2.2. Iontophoresis

**Figure 8:**
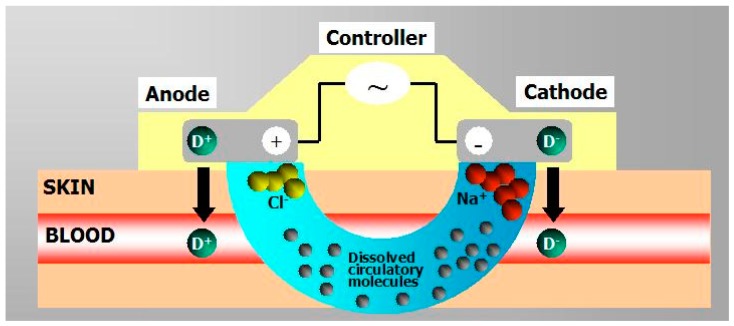
Schematic representation of an iontophoresis patch (Reprinted from [40] with permission. Copyright 2000 Elsevier).

Iontophoresis involves the application of physiologically acceptable electrical currents (0.1–1.0 mA/cm^2^) to drive charged permeants into the skin through electrostatic effects and make ionic drugs pass through the skin into the body by its potential gradient [5,20,58,68,69,70,71]. Unlike other transdermal enhancement methodologies, it acts mainly by involving a second driving force, the electrical potential gradient as companion to the concentration gradient across the skin since uncharged species can also be delivered through electroosmosis (Figure 8) [5,70].

**Figure 9:**
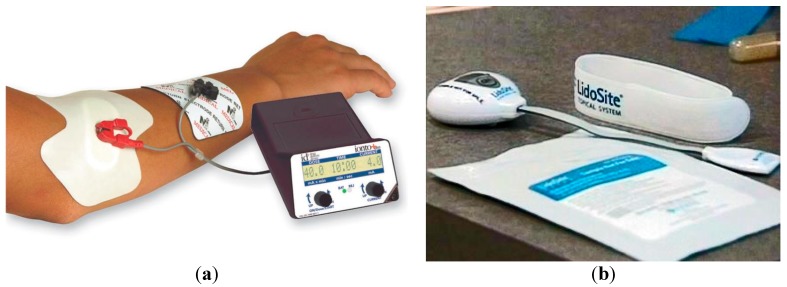
Commercially developed iontophoretic delivery systems: (**a**) Phoresor^®^ and (**b**) Lidosite^®^.

Phoresor^®^, Lidosite^®^, and E-trans^®^ are examples of three commercially developed iontophoretic delivery systems (Figure 9). The first approved commercial iontophoretic patch system was LidoSite^®^, which was developed to deliver lidocaine for fast dermal anaesthesia. The system was composed of a disposable pre-filled patch, re-usable battery-powered controller and a flexible interconnect module [20]. Iontophoresis has a minor effect on skin structure over short treatment periods due to the low-voltage nature of the applied electric current, when compared to electroporation [5].

Several factors affect iontophoretic TDD, including pH of the donor solution, electrode type, buffer concentration, current strength and the type of current employed [20,69,72,73]. The molecular size of the solute/drug is an important factor in determining its feasibility for successful iontophoretic delivery. The flux of smaller and more hydrophilic ions is faster than larger ions [72,73,74]. A plethora of studies correlating flux as a function of molecular weight have been conducted and it was found that the transport of compounds decreased with increase in molecular weight (chloride > amino acid > nucleotide > tripeptide > insulin) [22,72,75,76,77,78]. There is a linear relationship between the current and drug flux across the skin but the current is limited to 1 mA in order to facilitate patient comfort and consider safety concerns as with increasing current, the risk of nonspecific vascular reactions (vasodilatation) also increases [72]. Furthermore, the maximum time that the devices can be applied is 3 min, in order to prevent local skin irritation or burns. The maximum physiologically acceptable iontophoretic current is 0.5 mA/cm^2^ [79]. The current should be adequately high to provide a desired flux rate but it should not irritate the skin [80]. The use of continuous direct current (DC) can decrease the drugs flux due to its polarization effect on the skin [69]. In order to overcome this problem, pulsed current has been used [81]. Overall, only a limited number of studies have been carried out comparing pulsed direct current iontophoresis *vs.* continuous direct current iontophoresis. Recently, Kotzki *et al.* 2015 showed that pulsed iontophoresis of treprostinil significantly enhanced cutaneous blood flow compared with continuous iontophoresis [69]. The most common electrodes that are used in iontophoresis are aluminum foil, platinum and silver/silver chloride electrodes [73]. However, the preferred one is Ag/AgCl since it resists the changes in pH. In addition, the electrode materials used for iontophoretic delivery should be harmless to the body and flexible so as to be applied closely to the body surface [73].

The maximum molecular weight for iontophoretic delivery has not been extensively studied, although it is estimated that molecules with a molecular weight less than 12,000 Da may be successfully delivered across skin via iontophoresis [79]. In order to deliver molecules greater than 12,000 Da, an alternate means of overcoming the barrier properties of the stratum corneum must be sought. However, it was found that a small protein, cytochrome *c* (12.4 kDa) was delivered non-invasively across intact skin [82,83]. Afterwards, ribonuclease A, with isoelectric point of 8.64 (13.6 kDa), was successfully delivered across porcine and human skin [84]. More recently, it was shown that transdermal iontophoresis was also able to deliver biologically active human basic fibroblast growth factor (hbFGF; 17.4 kDa) in therapeutically relevant amounts corresponding to those used in clinical trials and animal studies [85,86].

The applications of iontophoresis can be classified into therapeutic and diagnostic applications. Iontophoresis has been used in various diagnostic applications e.g. diagnosing cystic fibrosis [87] and recently for monitoring blood glucose levels [88].The major advantage of iontophoresis in diagnostic applications is that there is no mechanical penetration or disruption of the skin involved in this approach [89,90].

### 4.3. Velocity Based Devices

**Figure 10:**
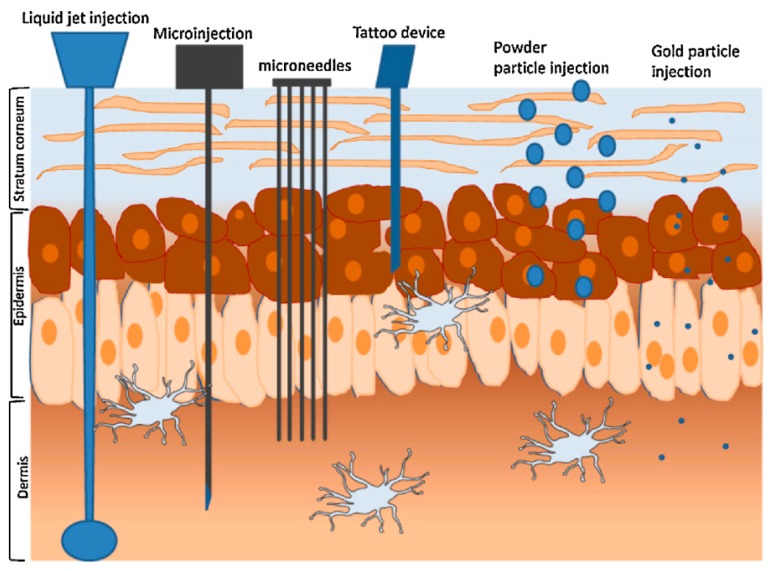
Methods for intradermal injection. (Reprinted from [93] with permission. Copyright 2005 Elsevier).

Velocity based devices, either powder or liquid jet injections, employ a high-velocity jet with velocities ranging from 100 to 200 m/s to puncture the skin and deliver drugs using a power source (compressed gas or a spring) [91]. The concept of jet injectors for use in drug delivery was first explored in the early of 1930s by Arnold Sutermesiter [11]. Since then, interest in this method of drug delivery has expanded significantly and two types of liquid jet injectors have been developed; single-dose jet injectors (disposable cartridge jet injectors) and multi-use-nozzle jet injectors (MUNJIs) [91]. Jet injections have been used for more than 50 years for parenteral delivery of vaccines, as well as small molecules, such as anesthetics and antibiotics [11]. A jet injector is a needle free device capable of delivering electronically controlled doses of medication which result in improved consistency of delivery and reduced pain for the patient (Figure 10) [48,92].

**Figure 11:**
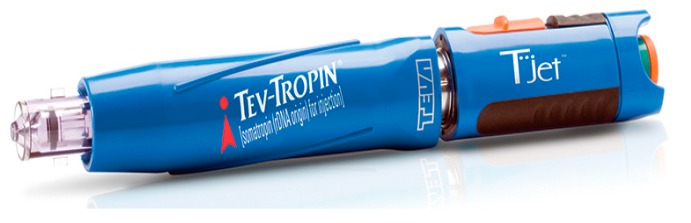
Commercially available jet injector Tjet^®^device.

Liquid-jet injectors propel liquid from a nozzle with an orifice diameter ranging from 50 to 360 μm, which is much smaller than the outer diameter of a standard hypodermic needle (810 μm for a 21G needle) [20,93,94]. The jet can deliver drug into different layers of skin e.g., intradermal (i.d.), subcutaneous (s.c.) or intramuscular (i.m.), by changing the jet velocity and orifice diameter [20]. The major advantage of using needle free devices relates to concerns regarding safe needle disposal and avoidance of accidental needle stick injuries [20]. However, the risk of cross contamination is not excluded, since splash back of interstitial liquid from the skin may contaminate the nozzle [95]. Therefore the use of multi-use nozzle jet injectors has been terminated and such devices are now only used for multi-dose drug delivery to the same individual, e.g., the Tjet^®^device which delivers somatropin (human growth hormone (hGH)) (Figure 11).

Powder jet injectors have an advantage over liquid jet injectors of delivering solid drugs or vaccines to the skin, so the stability of the formulation will be increased and the necessity for cold storage will be avoided, which simplifies transportation and reduces associated costs. Powder jet injectors may be formulated from nano-or micro-particles containing the active or lyophilised drugs and antigens [20,96]. Excellent bioavailability for a number of drugs has been reported but the intermittent pain and bruising caused to patients has restricted wide acceptance of jet injectors [91]. Regarding the levels of pain experienced by volunteers, some reports state no difference in the pain recorded when comparing jet injectors to conventional needle injections [97] but others have reported higher pain scores [98].

The basic design of solid jet injectors consists of compressed gas as the power source, drug loaded compartment containing solid drug formulation, and a nozzle to direct the flow of particles towards the skin [99]. By triggering the actuation mechanism, compressed gas expands and forces drug powder through a nozzle into the skin. Upon impacting on the skin, particles create micronsized holes and deposit in the stratum corneum or viable epidermis. The most important parameters that govern particle delivery across the stratum corneum are particle properties (size, density) and impact velocity e.g., for DNA vaccination, the particle size range should be between 0.5 and 3 µm [11].

### 4.4. Thermal Approaches (Lasers and Radio-Frequency Heating)

Thermal ablation is a method used to deliver drugs systemically through the skin by heating the surface of the skin, which depletes the stratum corneum selectively at that site of heating only, without damaging deeper tissues [49,100]. Many methods could be used to cause thermal ablation such as laser [101], radiofrequency [49,102], in addition to electrical heating elements [49]. In order to generate the high temperatures needed to ablate the stratum corneum without damaging the underlined epidermis, the thermal exposure should be short, so the temperature gradient across the stratum corneum can be high enough to keep the skin surface extremely hot but the temperature of the viable epidermis does not experience a significant temperature rise [100].

#### 4.4.1. Laser Thermal Ablation

Laser methodologies have been used in clinical therapies for the treatment of dermatological conditions such as pigmented lesions [101,103,104]. The main mechanism of laser thermal ablation of the skin is the selective removal of the stratum corneum without damaging deeper tissues, thus enhancing the delivery of lipophilic and hydrophilic drugs into skin layers [26,45,104,105]. Lasers ablate the stratum corneum by deposition of optical energy, which causes evaporation of water and formation of microchanels in the skin [106]. In addition, such approaches have been used to extract interstitial fluid for subsequent measurement of glucose levels in diabetic patients [49,101,103]. However, the degree of barrier disruption achieved is controlled by wavelength, pulse length, tissue thickness, pulse energy, tissue absorption coefficient, pulse number, duration of laser exposure and pulse repetition rate [48,58,107]. Baron *et al.*, 2003 demonstrated that pre-treatment with the laser followed by lidocaine cream was found to reduce the onset of lidocaine action to 3–5 min in human volunteers [106]. However, the structural changes in the skin must be assessed, especially at the higher intensities of laser employed that may be needed to enhance the transport of large molecular weight therapeutics [108,109].

#### 4.4.2. Radiofrequency (RF) Thermal Ablation

**Figure 12:**
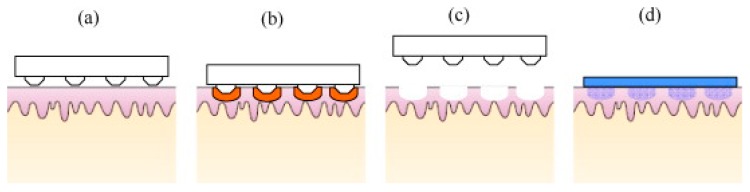
Schematic diagram of drug delivery using thermal ablation: (**a**) micro-electrodes are pressed against the skin, (**b**) skin is ablated via heating due to RF energy or resistive heating in the electrodes, (**c**) after removing the ablation device, (**d**) micropores formed. (Reprinted from [11] with permission. Copyright 2008 Elsevier).

Radiofrequency (RF) thermal ablation involves the placement of a thin, needle-like electrode directly into the skin and application of high frequency alternating current (~100 kHz) which produces microscopic pathways in the stratum corneum, through which drugs can permeate (Figure 12) [49,100]. Exposure of skin cells to a high frequency (100–500 kHz) causes ionic vibrations within the tissue which attempts to localize the heating to a specific area of the skin and thus ablate the cells in that region, resulting in drug transport across the skin [110]. This technology may enable transdermal delivery of a wide variety of hydrophilic drugs and macromolecules using a low-cost, fully disposable device [49].

### 4.5. Mechanical Approaches to Mediate Skin Permeation

The use of hypodermic needles, often associated with phobia, pain and the risk of needle-stick injuries have been used to overcome some of the delivery limitations often experienced when delivering macromolecular compounds [111,112]. Some innovative methodologies have been explored to overcome these issues and include the use of MN and tape stripping. These concepts will be described further below.

#### 4.5.1. Tape Stripping

Tape stripping is a simple method for removing the stratum corneum layer by repeated application of adhesive tapes [113]. The amount of *stratum corenum* removed by a single adhesive tape depends on many factors such as the thickness of the *stratum corenum*, the age of the patient, the composition and amount of lipid which varies depending on the anatomical site and finally, skin parameters such as transepidermal water loss (TEWL) and pH. In addition, other factors also affect the amount of stratum corneum removed by tape stripping, such as the force of removal of the tape from the skin and the duration of pressure on the skin [113,114]. Tape stripping is a robust and simple method. However, many parameters should be taken into consideration before and during the application of this procedure, such as the duration of pressure on the skin, in order to remove the stratum corneum homogeneously.

#### 4.5.2. Microneedle (MN) Arrays

**Figure 13:**
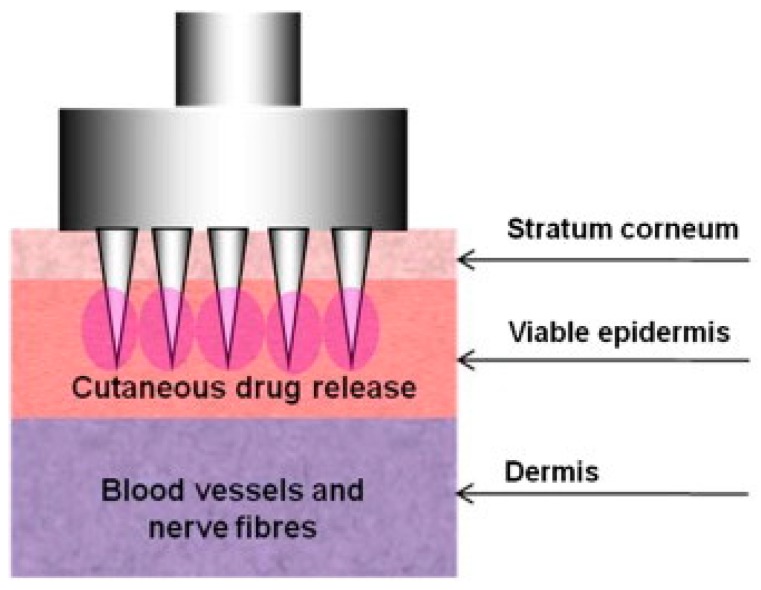
Schematic representation of the mechanism of action of a microneedle array device. The device perforates the stratum corneum (SC) providing direct access of drugs to the underlying viable epidermis, without reaching blood vessels and nerve fibres located in the dermis (Reprinted from [12] with permission. Copyright 2013 Elsevier).

MN arrays, minimally invasive drug delivery systems, were developed to overcome some of the disadvantages commonly associated with hypodermic needle usage and in order to address and improve patient compliance. MN arrays have the potential to be used as an alternative to hypodermic and subcutaneous needle technologies (Figure 13) [12,34,111,112]. MN technologies have been subject to intensive research and development efforts by both academic and industrial researchers with some devices currently in clinical development and others awaiting FDA approval [1,34]. Also the number of publications describing MN as novel minimally invasive devices for drug delivery purposes has grown exponentially in recent years [1,34,112,115]. As MN combine the ease of use of a transdermal patch with the effectiveness of delivery achieved using conventional hypodermic needle and syringes, they continue to elicit interest and investment [34,116].

MN are multiple microscopic projections typically assembled on one side of a supporting base or patch, generally ranging from 25 to 2000 μm in height [5,12], 50 to 250 μm in base width and 1 to 25 μm in tip diameter [20,112,117,118]. The needles should be of suitable length, width and shape to avoid nerve contact when inserted into skin layers [117,118,119]. They are usually designed in arrays in order to improve the surface contact with the skin and facilitate penetration of therapeutic molecules into the skin [112,120]. MN are designed to create transient aqueous conduits across the skin, thereby enhancing flux of the molecules ranging from small hydrophilic molecules such as alendronate [52] to macromolecules, including low molecular weight heparins [4,121], insulin [122] and vaccines [123], in a pain-free manner [112,124]. Besides the aspect of pain-free delivery, there are many other advantages of MN technologies, such as: the fact that they do not cause bleeding [125]; eliminate transdermal dosing variability of small molecules [45,126]; only minimal introduction of pathogens through MN-induced holes [124,127]; potential for self-administration [1,128]; the potential to overcome and reduce instances of accidental needle-sticks injuries and the risk of transmitting infections [12,112], in addition to the ease of MN waste disposal [11,112].

As conceded previously in this review, one of the most attractive applications of MN arrays is to use them in vaccination and indeed, self-vaccination strategies. The skin contains high concentrations of adaptive and innate immune cells including macrophages, Langerhans cells, and dermal dendritic cells. To date, only oral typhoid vaccine is approved for self-administration in patients’ homes [129]. Injecting vaccines into the epidermis or dermis is immunologically superior to injecting into the muscle where much lower populations of immune cells reside and this MN approach therefore offers excellent amplification potential for the desired immune response [21,130]. As a result, the dose required to vaccinate through the skin via MN will be much lower than that require dosing of a conventional needle and syringe injection into the muscle. Vaccine delivery via the skin offers easier and painless administration. Moreover, these MN vaccination devices can be manufactured inexpensively [5,34,112].

**Figure 14:**
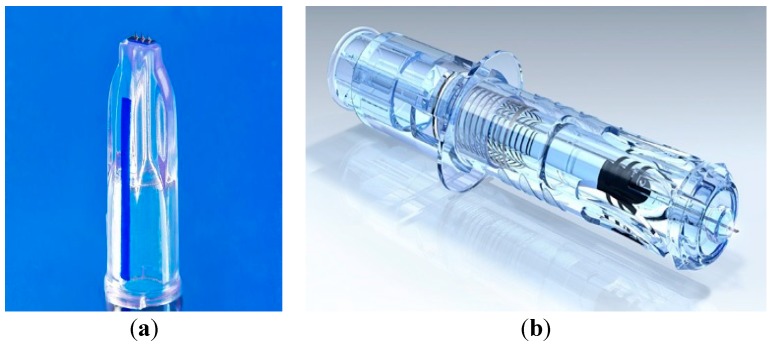
Current commercial MNs-based products (**a**) Intanza^®^ and (**b**) MicronJet^®^.

The first two commercially marketed MN-based products are Intanzia^®^ and Micronjet^®^ which are based on metal and silicon MN, respectively (Figure 14) [131]. Intanza^®^ is the first influenza vaccine that targets the dermis, a highly immunogenic area. It was developed and licensed by Sanofi Pasteur MSD Limited and is being marketed in two strengths; Intanza^®^ 9 µg for adults aged between 18 and 59 years and Intanza^®^ 15 µg for adults of 60 years and above. The Intanza^®^ influenza vaccine system has a needle length of 1.5 mm [132]. MicronJet is a single use, MN-based device for intradermal delivery of vaccines and drugs. It was developed and licensed by NanoPass.

Several companies have been working towards the development of MN-based drug or vaccine products, including 3M, Clearside Biomedical, NanoPass Technologies, Corium International, TheraJect, Circassia, Radius Health, Lohmann Therapeutic Systems (LTS) and Zosano Pharma. Zosano has developed a transdermal patch consisting of an array of titanium MN coated with parathyroid hormone (PTH) (20 to 40 μg) attached to an adhesive patch and applied via a reusable applicator across the skin [1,133]. A second study involving the Zosano titanium MN patch system was carried out by Ameri *et al.* 2014 to evaluate the feasibility of titanium MN usage to deliver recombinant human growth hormone (rhGH) [126]. In this study, it was found that the bioavailability of the rhGH MNpatch and the current subcutaneous injection products (Norditropin^®^) were similar which indicates that this MN product can be used as a patient-friendly alternative to subcutaneous injection of Norditropin^®^ [126,133]. The 3M Microneedle Technologies (MTS) has developed coated MN to deliver water-soluble, polar and ionic molecules, such as lidocaine, through the skin. This system has successfully delivered drugs to the skin within seconds and provide rapid onset of local analgesia (~1 min) which facilitates routine or emergency procedures [51,134].

**Figure 15:**
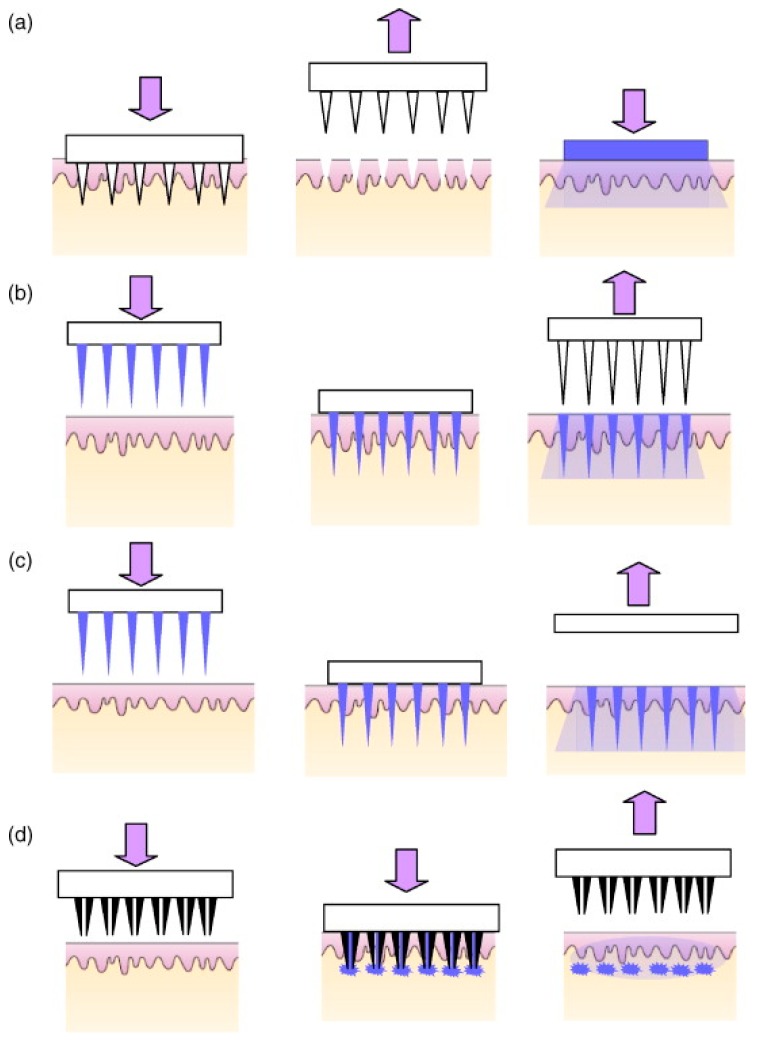
A schematic representation of four different MN application methods used to facilitate drug delivery transdermally. (**a**) Solid MNs for increasing the permeability of a drug formulation by creating micro-holes across the skin; (**b**) Coated MNs for rapid dissolution of the coated drug into the skin; (**c**) Dissolvable MNs for rapid or controlled release of the drug incorporated within the microneedles; (**d**) Hollow MNs used to puncture the skin and enable release of a liquid drug following active infusion or diffusion of the formulation through the needle bores. (Reprinted from [11] with permission. Copyright 2008 Elsevier).

**Figure 16:**
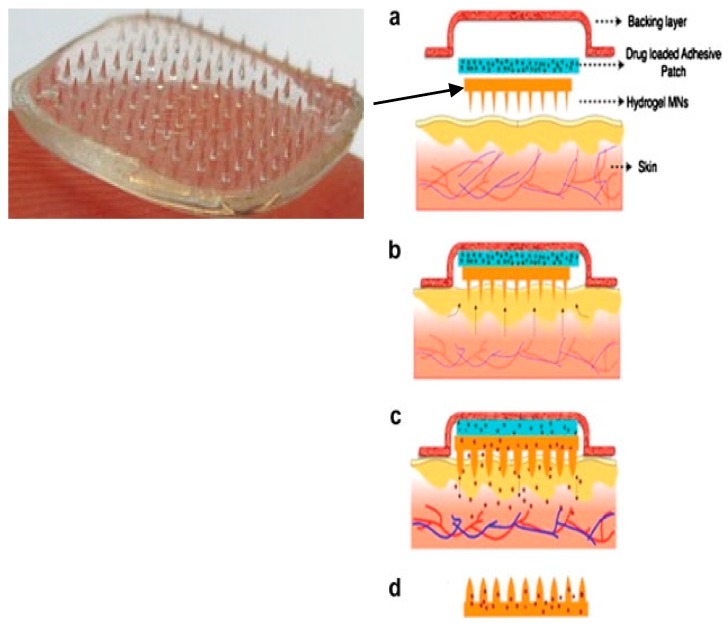
Novel hydrogel-forming MN facilitate controlled transdermal drug delivery. (**a**) An expanded view of the backing layer, drug-loaded adhesive patch and solid crosslinked hydrogel MN array which constitutes an integrated hydrogel MN patch; (**b**) Application of the integrated hydrogel MN patch to the skin surface; (**c**) Diffusion of water into the MN array leading to controlled swelling of the arrays and diffusion of drug molecules from the adhesive patch through the hydrogel conduit; (**d**) Intact hydrogel MN arrays following removal from the skin. (Reprinted from [12] with permission. Copyright 2013 Elsevier).

The shape and geometry of MN is critical during design and fabrication [22,135,136,137]. The needles must be capable of inserting into the skin without breaking and the needles should be of suitable length, width and shape to avoid nerve contact and create efficient pathways for the delivery of small drugs, macromolecules and nanoparticles, as well as for fluid extraction, depending on the objectives of each device [115,117,119,138]. The elastic properties of human skin may prevent effective MN penetration by twisting of the skin fibers around the needles during application, particularly in the case of blunt and short MN [117]. To date, many papers have described the fabrication of various MN from different materials using various micro-moulding processes or other methods, such as lasers [112,139,140]. Generally, there are four strategies of TDD using MN (Figure 15) [22,123]. These are solid, coated, dissolvable and hollow MN. A novel fifth MN-type, namely hydrogel MN have garnered much interest in the recent past and are presented in Figure 16.

(1) Hollow MN are used to deliver drug solutions via the “poke and flow” method; which involves insertion of the MN into tissue and then a drug solution can be transported through the bore of the MN in similar fashion to a hypodermic needles [141,142] but hollow MN usually require very precise and high cost manufacturing technology [111]. Passive diffusion of the drug solution may occur through the MN, but active delivery allows for more rapid rates of delivery. Active delivery requires a driving force, a syringe can be used to drive the solution through the MN into the tissue but some studies have combined the MN systems with a pump or pressurised gas [143,144].

(2) “Poke and patch” mainly for solid MN by piercing the upper layers of the skin with solid MN and creating microchannels followed by application of a drug formulation (e.g., patch, gel) at that site piercing [5,112]. The skin pretreatment creates micro-conduits in the skin, thereby enhancing flux of the molecules through the skin.

(3) “Coat and poke” by piercing the skin with drug coated solid MN, which solve the problem of two-step application and provide extremely quick drug delivery [111,145]. Delivery from coated MN was found to be attractive especially for high molecular weight molecules [146]. However, drug delivery is limited due to the small dimensions of the MN shaft and tip [146,147,148].

(4) “Poke and release” for dissolving/porous/hydrogel forming MN through which drug will diffuse into systemic circulation (Figure 16). The materials from which the MN are produced act as drug depots holding the drugs until the trigger for release occurs, *i.e.*, dissolution in the case of dissolvable MN or swelling in the case of hydrogel MN [22,131,149]. This strategy eliminates the need for sharps disposal, and the possibility of accidental reuse of MN. Moreover, dissolvable MN patches have been reported to successfully deliver both small (MW 500 Da) and macro molecules (MW 500 Da) in “poke and release” approaches [25,26].

A wide variety of MN types and designs have been shown to be effective for the transdermal delivery of a diverse range of molecules, both *in vitro* and *in vivo* [10,12]*.* The potential now exists to greatly expand the range and types of drugs that can be delivered effectively across the skin. This will significantly enhance the value of the transdermal delivery market and will be increasingly important over the coming years as the number of new drugs of biological origin continues to increase. Future studies will be needed to address potential regulatory concerns over the use of MN devices, as well as focusing on the design and development of processes to enable a low cost, efficient means for MN mass production. A number of other physical approaches such as sonophoresis, electroporation, ultrasound and iontophoresis have been combined with MN in order to enhance permeation of drugs. Kolli *et al.*, 2012 determined that the transdermal delivery of Prochlorperazine Edisylate was significantly enhanced when MN were used in conjunction with iontophoresis [150]. Moreover, the delivery of ropinirole hydrochloride by MN and iontophoresis was significantly higher compared to modulated iontophoresis alone [151].

## 5. MN Overcome Many of the Limitations Associated with Other TDD Methodologies

Various limitations associated with each of the outlined TDD approaches have been documented throughout this review. To this end, MN methodologies may prove an efficacious, cost-effective and patient friendly alternative in choosing a TDD system for delivery of a host of drug molecules. With this in mind, some of the advantages of MN approaches over other TDD systems are outlined below.

As a novel and minimally invasive approach, MN are capable of creating superficial pathways across the skin for small drugs, macromolecules, nanoparticles, or fluid extractions to achieve enhanced transdermal drug delivery [152].Their sharp tips are short enough to limit contact with skin nerves, thus preventing pain sensation [125] and they are narrow enough to induce minimal trauma and reduce the opportunities for infections to develop following insertion [127]. This method combines the efficacy of conventional injection needles with the convenience of transdermal patches, while minimizing the disadvantages of these administration methods [152,153]. Moreover, MN can be manufactured using various types of material e.g., polymers, metal or silicon. Biocompatible and biodegradable polymers can be safely applied to the skin and are generally cost-effective. Various polymeric materials such as poly-l-lactic acid, poly-glycolic acid, poly-carbonate, poly-lactic-*co*-glycolic acid (PLGA), poly-dimethylsiloxane, a copolymer of methyl vinyl ether and maleic anhydride, carboxymethyl cellulose, maltose, dextrin and galactose have all been used to fabricate MN [139]. MN can also deliver a wide range of drugs ranging from small molecular weight e.g., ibuprofen [124] to high molecular weight e.g., ovalbumin compounds [131]. Immunization programs in developing countries via MN could be applied with minimal medical training and with lower associated costs. In addition, MN arrays have recently been used as an alternative approach in the minimally-invasive sampling of fluids from patients, without causing pain or bleeding in the advancement of novel therapeutic drug monitoring systems [12]. Although MN technologies show tremendous promise in the field of TDD, there are still relatively few FDA approved MN devices. A number of challenges which must be addressed before MN become widely available include scale up manufacture to industrial levels which will require considerable planning and standardization. In addition, MN device regulatory considerations must be established and addressed. These issues may include but are not limited to, issues surrounding product sterility; the potential for accidental reuse of certain MN modalities (e.g., solid MN), appropriate packaging and manufacturing aspects and the potential for undesired immunological effects. These must all be addressed before MN devices receive widespread approval. Moreover, the choice of appropriate biomaterials for preparation of MN is limited due to lack of mechanical strength, poor control of drug delivery, and limitation of drug loading dose [154,155].

## 6. Conclusions

In conclusion, the TDD sector continues to grow and develop with rapid expansion in fundamental knowledge feeding industrial development. In time, it is hoped that technological advancements in TDD will lead to enhanced disease prevention, diagnosis and control, with concomitant improvement in health-related quality of life for patients worldwide. To this end, this review has charted the development of numerous novel TDD methodologies, highlighting the advantages and disadvantages of each approach. Due to the exponential growth in investment and interest in MN technologies and the numerous associated advantages of this approach, particular attention was paid to this TDD system.
